# Atomic dispensers for thermoplasmonic control of alkali vapor pressure in quantum optical applications

**DOI:** 10.1038/s41467-019-10158-4

**Published:** 2019-05-24

**Authors:** Kristina R. Rusimova, Dimitar Slavov, Fabienne Pradaux-Caggiano, Joel T. Collins, Sergey N. Gordeev, David R. Carbery, William J. Wadsworth, Peter J. Mosley, Ventsislav K. Valev

**Affiliations:** 1 Centre for Photonics and Photonic Materials, University of Bath, Bath, BA2 7AY UK; 2 Centre for Nanoscience and Nanotechnology, University of Bath, Bath, BA2 7AY UK; 3 Institute of Electronics, Bulgarian Academy of Sciences, Sofia, 1784 Bulgaria; 4 Department of Chemistry, University of Bath, Bath, BA2 7AY UK

**Keywords:** Atom optics, Nanophotonics and plasmonics, Quantum optics

## Abstract

Alkali metal vapors enable access to single electron systems, suitable for demonstrating fundamental light-matter interactions and promising for quantum logic operations, storage and sensing. However, progress is hampered by the need for robust and repeatable control over the atomic vapor density and over the associated optical depth. Until now, a moderate improvement of the optical depth was attainable through bulk heating or laser desorption – both time-consuming techniques. Here, we use plasmonic nanoparticles to convert light into localized thermal energy and to achieve optical depths in warm vapors, corresponding to a ~16 times increase in vapor pressure in less than 20 ms, with possible reload times much shorter than an hour. Our results enable robust and compact light-matter devices, such as efficient quantum memories and photon-photon logic gates, in which strong optical nonlinearities are crucial.

## Abstract (web summary)

Robust and fast control of the atomic vapor pressure in alkali vapor cells would greatly extend their use for many quantum technologies. Here, the authors exploit plasmonic absorption in a cell coating containing gold nanoparticles to control the vapor pressure with milliseconds response time.

## Introduction

Alkali metal vapors in confined geometries constitute a fertile ground for quantum optical technology^1^, both as isolated atoms and as coherent ensembles of atoms^2^. Alkali metal atoms can be viewed as one electron systems, due to the presence of a single electron in the outer shell. The resulting sharp resonances find applications in atom cooling, precision spectroscopy and in the frequency stabilization of lasers on atomic transitions^3^. Associated Λ-type systems serve as a basis for atomic clocks^4,5^ and magnetometry (e.g., for cardiograms and encephalograms)^6–10^, using coherent population trapping (CPT). The CPT effect also leads to electromagnetically induced transparency (EIT). In turn, the sharp variation of refractive index in the narrow frequency region of EIT can slow down light pulses, effectively trapping them in a vapor cell^11^. Mapping optical quantum states into coherent behaviors of alkali-metal ensembles, for example atomic spin waves, enables quantum memory^1,12,13^, as required for synchronising single-photon sources and long-range quantum cryptographic networks^14^.

In alkali-metal atoms, the Rydberg state corresponds to an excitation of the outer shell electron to a state with high principal quantum number *n*. In such states, the effective dipoles are enlarged by a factor of *n*^2^ compared to ground-state atoms. Huge interactions between individual atoms arise, due to overlapping wave functions, and allow controlled entanglement of the atomic ensembles^15^. Such systems can induce strong nonlinearity even at the single-photon level; absorbing a photon into a Rydberg state causes the energy levels of all the atoms within the ensemble to shift, preventing the absorption of another photon, i.e., the system becomes transparent. This behavior is known as Rydberg blockade and it enables various quantum gates or switches^2,16–19^, it has the potential to form quantum logic networks if it can be harnessed in a robust and reliable package. In particular, it allows a system to be transparent to a single photon but absorbing for a photon pair, which is the essence of nonlinearity^20,21^. In addition, the energy level structure of Rb provides the basis for almost-degenerate two-photon excitation near 780 nm; the resulting off-resonant enhancement boosts nonlinear optical effects such as four-wave mixing^22^. The latter can yield squeezed states with reduced amplitude or phase uncertainty, giving rise to a nonclassical enhancement of measurement precision in interferometers^23,24^, (useful for inertial sensors and space-based gravity wave detectors), quantum limited amplifiers^25^, and quantum delay lines^23^.

Underlying all these applications is the ability to manipulate the vapor pressure of atomic ensembles within a confined geometry. The resulting optical depth is a key parameter in determining performance metrics such as the strength of the optical nonlinearity^2^, the efficiency of an alkali-based quantum memory, and the likelihood of exploiting effective photon-photon interactions within the Rydberg blockade volume. In view of the alkali-metal’s reactivity, adsorption to the cell walls typically limits the optical depths achievable. As a passive measure, various optical coatings have been applied to prevent the atoms from reaching the walls and to preserve their quantum states^26^. Active control of the optical depth is achieved with conventional Light-Induced Atomic Desorption (LIAD)^27–29^. Conventional LIAD proceeds from low-intensity, preferentially blue-ultraviolet illumination, via non-thermal mechanisms^30–32^, or from high-intensity, preferentially infrared illumination, via direct heating/vaporization of alkali-metal structures^28^. So far, the best control of optical depth was achieved with feedback loops on LIAD in cells^29^ and hollow-core fibers^27,28^. However, it is very slow-desorbing atoms take tens of seconds, requiring hours of recovery time and imposing correspondingly long intervals between successive experiments^33–35^.

Here, we report a new coating for alkali-metal vapour containers, based on plasmonic nanoparticles (NPs). Illuminating the coated vapor cells with visible light dispenses Rb atoms >1000 faster than previously reported, in a reproducible manner. We exploit the plasmonic absorption of the NPs to efficiently transduce electromagnetic energy into localized thermal energy. We report on the photothermal behavior of plasmonic gold NPs in the presence of highly reactive alkali vapor that is capable of forming aurides and of disrupting the molecular bonds in the coating. The photothermal process causes desorption of Rb atoms near the NPs, whose small size is also responsible for the fast response time. Contrary to conventional LIAD^33^, the Rb desorption per photon, as a function of photon energy, does not depend linearly on the photon energy of the incident light; rather it follows the trend of the plasmonic absorption resonance. In addition, the Au NP coating shows no permanent magnetism and therefore does not influence the coherence of the Rb atoms.

## Results

### Atomic vapor cell coating based on plasmonic nanoparticles

**Fig. 1:**
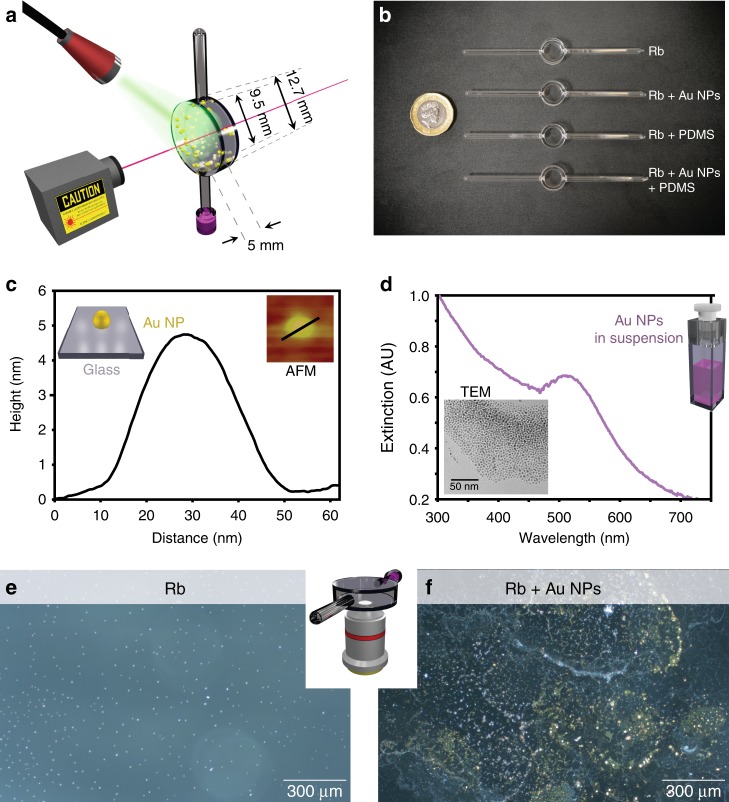
A coating for atomic vapour cells, based on plasmonic nanoparticles. **a** Schematic diagram of our experiment. **b** A photograph of the fabricated coated and reference cells. **c** Line profile of the atomic force microscopy (AFM) image (in inset) of a 5 nm Au nanoparticle (NP) deposited on a glass substrate. **d** Extinction spectrum of the Au NPs in hexane solution. The inset shows transmission electron microscopy (TEM) image of the Au NPs deposited on a grid. **e** and **f** Dark field optical microscopy images of the interior wall of the reference and Au-coated cells, respectively. Images were acquired through a flat cell window as illustrated in the inset, with a x5 objective

Figure 1a illustrates the crux of our experiment. The inside of a Rb vapor cell was coated with gold nanoparticles, whose surface plasmon resonance (SPR) was excited by illumination from a 532 nm (green) laser. The excited SPR causes increased light absorption by the NPs, which then transduce light energy into heat. The heat causes Rb atoms in the vicinity of the NPs to desorb from the walls and increase the atomic vapor pressure inside the cell. The D_2_ transition of Rb was probed with a 780 nm laser to measure absorption by mobile Rb atoms. Due to the very small volume of the nanoparticles, the heating process and associated Rb desorption are much faster than previously reported^33,34^. Hence, rapid increases in the absorption, as well as higher optical depths, are measured in our coated cells.

For our experiments, we prepared four Rb filled vapor cells of identical dimensions, as illustrated by the photograph in Fig. 1b. In order to investigate the effects of the cell coating, first we prepared an uncoated reference cell. Next, a cell was coated with Au NPs, capped with octadecylamine (ODA)^35^. The ODA molecules serve as linkers between the cell walls and the Au NPs. In order to preserve the atomic state of Rb, in quantum optics technology, the cell walls are often coated with polydimethylsiloxane (PDMS). Consequently, to demonstrate the benefits of our Au NP coating in such technologies, we prepared two more cells: one had only PDMS coating, and the other had both PDMS and ODA-capped Au NPs coating.

The Au NPs diameter was characterized by atomic force microscopy (AFM), transmission electron microscopy (TEM), and UV-visible spectroscopy, see Fig. 1c, d. Figure 1c shows the line profile of an AFM topography (in inset) of a microscope glass slide that was coated with ODA-capped Au NPs. The height of the line profile suggests that the synthesized NPs are 5 nm (±1 nm) in size. This is in good agreement with the extinction spectrum in Fig. 1d. This spectrum was obtained from a dilute solution of the ODA-capped Au NPs in hexane, with the hexane background subtracted. The spectrum shows a clear plasmon resonance centered at 516 nm, which corresponds to NPs of <8 nm diameter. Moreover, Fourier transform analysis of the TEM image in Fig. 1d indicates that the NPs have an average diameter of 5 nm (±2 nm). Gold nanoparticles from the characterized batch are then deposited inside two vapor cells. This size of the obtained nanoparticles is typical for the technology of Amine-caped Au-NPs preparation^35–38^.

Figure 1e shows a dark field microscopy from the inside wall of the reference vapor cell (without Au NPs). The experimental configuration for taking the microscope image is presented in the inset. In the micrograph, the pale blue dots correspond to scattering from Rb crystalline particles, condensed on the inside window. By contrast, Fig. 1f shows a similar dark field micrograph from the vapor cell with Au NPs. The latter can be seen as green patches and yellow aggregates. These Rb cells are then placed within the optical setup.

### Dramatic increase in vapor pressure upon light illumination

**Fig. 2:**
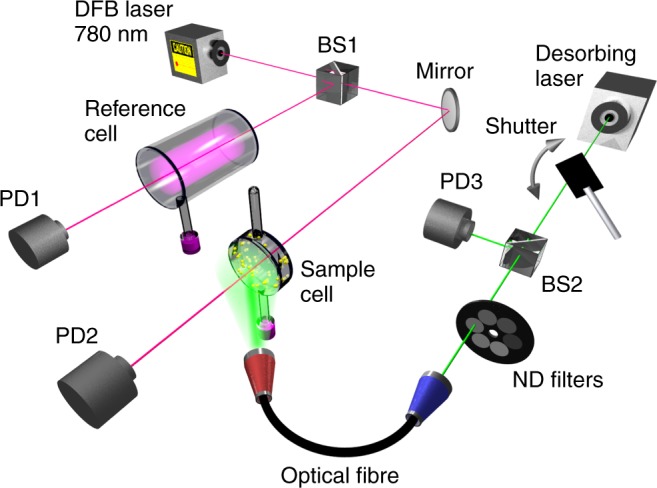
Schematic diagram of the experimental setup. DFB distributed feedback laser, BS1 and BS2 beam splitters, PD1, PD2, and PD3 photo detectors, ND filters neutral density filters. The DFB laser current is externally modulated to scan the D_2_ line of Rb

The optical setup used to study the Rb desorption is sketched schematically in Fig. 2. The 780 nm distributed feedback (DFB) laser was externally modulated to scan over the full frequency range of the Doppler-broadened D_2_ absorption line of Rb. The DFB laser beam was split, deflecting some of the light towards photodiode 1 through an off-the-shelf reference cell filled with Rb in a natural mixture of isotopes. The rest of the beam was guided through the Au NP coated cell and the Rb absorption spectrum was monitored at photo diode 2. For the desorbing illumination, we used four different laser wavelengths: 406 nm, 430 nm, 532 nm, and 655 nm. In all cases, the light was modulated with an electronically controlled shutter. Subsequently, using beam splitter 2, part of the beam was directed to photo diode 3, which recorded the shutter duty cycles. The desorbing laser light was further attenuated using a neutral density filter wheel and it was then coupled into an optical fiber, used to illuminate the vapor cell.

**Fig. 3:**
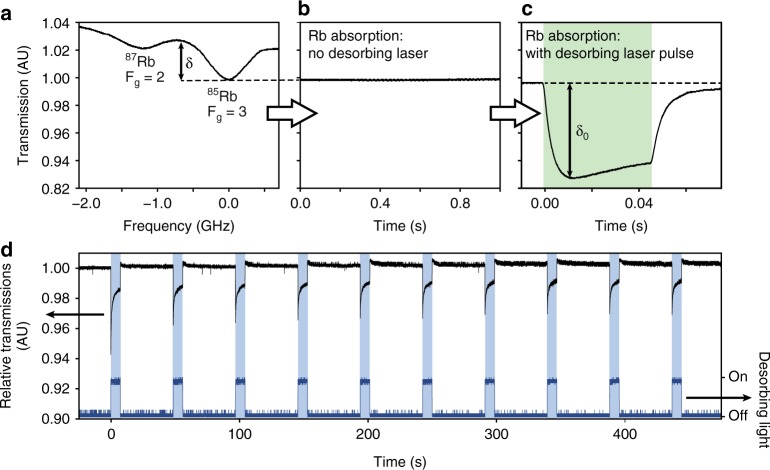
Strong and reproducible increase in atomic cell vapor pressure upon illumination. **a** Doppler-broadened absorption spectrum of Rb D_2_ line, obtained upon modulating the frequency of the DFB laser, at cell temperature of 17 °C. **b** Stabilizing the laser modulation at the F_g_ = 3 transition of ^85^Rb yields a constant light intensity signal. **c** A strong increase of absorption is observed upon illuminating the cell with 532 nm, 32 mW/cm^2^ light for 45 ms. **d** For repeated illuminations with 406 nm, 31,5 mW/cm^2^ light for 7 s (shutter in Fig. 2), the increased absorption is clearly reproducible. The initial peaks are due to bursts of desorbed atoms that subsequently migrate throughout the cell volume

Figure 3 demonstrates that illuminating the nanoparticles, with desorbing laser light, dramatically increases the vapor pressure in the cells; moreover, the process is highly reproducible. Figure 3a shows the Doppler-broadened D_2_ absorption line of the natural Rb mixture inside the Au NP coated vapor cell (recorded with PD2). Two pronounced dips can be seen, corresponding to the ^87^Rb (and ^85^Rb) F_g_ = 2 (and F_g_ = 3) transition lines, respectively. The depth of ^85^Rb F_g_ = 3 transition line is indicated as δ. The DFB laser frequency is then stabilized at the maximum absorption of the F_g_ = 3 line, as shown in Fig. 3b. The recorded trace clearly demonstrates the stability of the DFB laser over an entire second. This period is much larger than the desorbing light pulses which, for 532 nm, 31.5 mW/cm^2^ laser light, were 45 ms in duration. In response, the absorption by Rb vapor inside the cell rapidly increases, as shown in Fig. 3c, where the maximum is reached in ~14 ms. This increase is evidenced by the depth of the Rb absorption line δ_0_, which is much larger than δ. It should be noted that the fast decrease of the Rb absorption line, here <20 ms, is equally important, for instance in magneto-optical traps^39^. Indeed, once such a trap is constructed, the remaining free atoms are disruptive and need to be quickly extracted from the vicinity of the trap. In our case, the fast increase/decrease is observed at various desorbing laser wavelengths and is highly reproducible.

In Fig. 3d, we show ten consecutive illuminations of the vapor cell with 31,5 mW/cm^2^ but at a different desorbing laser wavelength (406 nm). For each desorbing light pulse, a sharp increase δ_0_ can be seen, on illumination. As the shutter closes, a sharp decrease of light absorption occurs. This behavior is clearly reproducible over all light pulses. Compared to Fig. 3c, the longer duration (7 s) of the light pulses in Fig. 3d allows us to observe the behavior of the Rb vapor in more detail, during illumination. The initial spike in the absorption depth is followed by a gradual decrease, due to the depletion of the Rb atoms in the coating. At the end of the desorbing light pulse, the absorption depth decreases, together with the Rb vapor density, towards the thermal equilibrium. After each illumination, the coating requires time to recover and load with Rb atoms. For each consecutive light pulse, the initial spike in absorption depth diminishes. This is because, over time, the Rb atoms migrate through the cell volume and aggregate in the cold parts of the cell, for example the two stems outside the region illuminated by desorbing light. For the rest of the experiments in this paper, the coating was allowed to recover for 1 h before each illumination with the desorbing light. In a miniaturized cell, this recovery time will be much shorter.

### Rb desorption yield and NP extinction

**Fig. 4:**
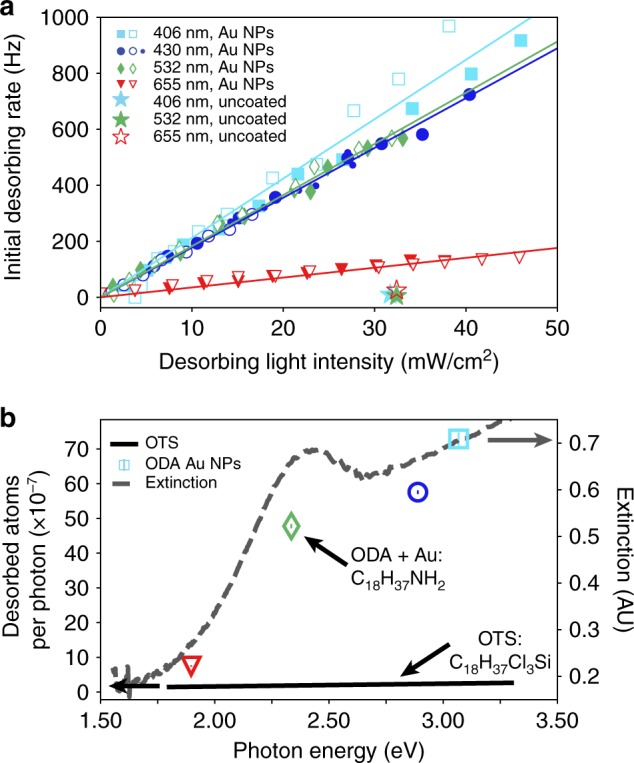
Rb desorption yield follows the extinction spectrum profile of the Au NPs. **a** The initial desorption rate of Rb from the walls of the Au NP coated vapor cell exhibits a linear dependence on the desorbing light intensity, for four wavelengths: 406 nm, 430 nm, 532 nm, 655 nm. Cell temperature 17 °C. Control experiments with the uncoated reference vapor cell (marked with stars) show no increase in desorption upon illumination with light of all four wavelengths. The control experiments were offset from 0 for clarity. Filled and unfilled data points correspond to data taken on different days. **b** Calculated yield (Rb desorption per photon) of the desorption process for each wavelength and comparison with calculation for an OTS coated vapor cell from ref. ^33^ cell temperature 25 °C. Gray dotted line: comparison with Au-capped ODA extinction spectrum as a guide to the eye

Figure 4 indicates that the Rb desorption is caused by plasmonic heating of the gold nanoparticles. To establish this, we studied the initial rate of Rb desorption vs. the desorbing light intensity at four different wavelengths, as shown in Fig. 4a. For each wavelength there is a linear dependence of desorption rate on intensity; the gradient of the relationship is smallest for 655 nm, i.e., where the Au NPs have low absorption (Fig. 1d) and so generate the least heat. For reference, the uncoated vapor cell shows no measurable increase in δ_0_ (see star symbols) in the absence of heat generating NPs. Two separate data sets (full and empty symbols) demonstrate that the data are robust.

To further emphasize the role of plasmonic heating, we perform a linear fit to the data in Fig. 4a and estimate the yield (γ) of the desorption process per photon, at each wavelength. The calculation procedure is detailed in the Supporting Information. Figure 4b shows the calculated values of γ, measured in desorbed atoms per photon, as a function of photon energy. For clarity, the four wavelengths in Fig. 4a are represented by symbols of identical shape and color to the ones in Fig. 4b. Because our coating consists of both Au NPs and linker molecules (ODA), it is important to differentiate between their respective effects on the Rb desorption. This can be achieved by comparing our results to those from Octadecyltrichlorosilane (OTS) molecules^33^, which are very similar to ODA. The OTS data are obtained at 25 °C and are shown as a black line in Fig. 4b, from which it is immediately obvious that the efficiency of the Au NP coating is far superior to that of the OTS coating. Moreover, the desorption efficiency of OTS depends linearly upon photon energy^33^, whereas this is not the case for our coating. In fact, the shape of our data is reminiscent of the measured extinction spectrum in Fig. 1d, here plotted as function of photon energy as a gray dashed line. It should be noted that the Au NPs spectrum in solution is red-shifted in comparison to that of Au NPs in vacuum^40,41^. This spectral shift contributes to the difference between the symbols and the gray line.

### Combination with conventional polymer coating

**Fig. 5:**
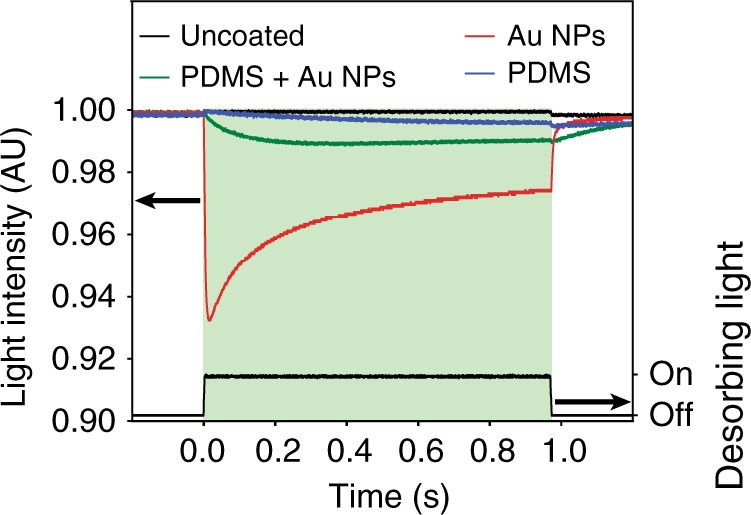
Compatibility of Au NP coating with conventional polymer coatings. The Au NP coating is compatible with the anti-relaxation properties of PDMS that are key for quantum optical applications. The increased absorption of Rb was recorded for all four vapor cells when illuminated with a green laser pulse at 532 nm, at 17°. The uncoated reference cell (in black) shows no increase in Rb absorption. The Au NP only coated cell (in red) shows the largest increase in Rb absorption, and it quickly returns to the initial absorption depth after the desorbing light pulse. The PDMS only coated cell (in blue) undergoes a small increase in absorption during the desorbing light pulse, which slowly decreases, afterwards. By comparison, the cell coated with both PDMS and Au NPs (in green) demonstrates faster and more than two-fold increase in absorption

To illustrate the advantage of using our Au NPs in potential quantum photonics applications, we applied it in conjunction with PDMS—a standard anti-relaxation coating for these applications. Figure 5 shows the behavior of the Rb absorption line when illuminated for 1 s with a 532 nm laser pulse; data are shown for each of the four cells presented in Fig. 1b. The traces were recorded in a similar fashion to that in Fig. 3c. It is immediately obvious that for this desorbing wavelength, the cell coated with Au NPs and PDMS (green line) undergoes a clear increase in Rb absorption, compared to the PDMS only cell—following a much faster initial increase, δ is doubled after 1 s, i.e., by the end of the light pulse. This result is very significant, given the suboptimal position of the NPs with respect to the PDMS. Indeed, the purpose of our coating is to interact with Rb atoms and the thick PDMS layer effectively acts as a separator. Even better coating performance can therefore be expected from, for instance, dispersing the Au NPs within the PDMS.

The reach of the thermal processes discussed so far is limited by the thermal conductivity of the system. Within the smaller volumes of the plasmonic excitation, Rb desorption can also result from hot electron-induced photodissociation of Rb from the ODA molecules, by transfer of a hot electron into the anti-bonding orbital of the atom^42–45^.

Our study is the first to investigate the triple system (Au + Octadecylamine + Alkali atoms), in the steady state and upon excitation of the plasmon resonance. Although ODA-coated-Au nanoparticles are well-known, their properties and stability have never been tested in the presence of highly reactive alkali atoms. There are very good reasons to doubt this stability.

Separately, the (Alkali atom + Amine)^46,47^, (Alkali atom + Gold)^48,49^, and (Amine + Gold)^35–38^ have all been investigated, for alternative applications. The results show that thermally excited alkali atoms and gold form aurides, whose properties can greatly differ from gold. Moreover, amines form bonds with the alkali atoms, whereby their reactivity significantly decreases, for longer hydrocarbon-tails. In our system, ODA chains on the spherical NP-surface do not form an atom-tight coating. Consequently, Rb atoms can penetrate to the Au bonded amine head and can influence the strength of this bond. Rb can also attach to the tail of the ODA molecule.

To demonstrate the long-term stability of the ODA-Au-NP coating, we compare the case where it is exposed directly to the alkali atom vapor with the case where it is protected by a PDMS layer. PDMS was chosen because of its well-established desorption and anti-relaxation properties. We observe that the unprotected ODA-Au-NPs coating displays excellent stability, presenting no visible changes 20 months after its production.

Our coating can further be optimized, for example using layer-by-layer methods^50^. Further optimization of Au-NPs density, size, shape, aspect ratio, ordering, and design (core-shell)^51^ can tailor the plasmonic particles to specific quantum optical applications.

## Discussion

Although bulk gold presents well-established diamagnetism, gold nanoparticles can have curious magnetic properties. Ref. ^52^ summarizes significant examples and presents a range of possible physical mechanisms for the origin of a permanent magnetic moment in Au NPs. In our experiments, such a magnetic moment would constitute a source of stray magnetic field at the cell wall and may potentially influence the magnetic polarization and coherence of Rb atoms, limiting applications.

**Fig. 6:**
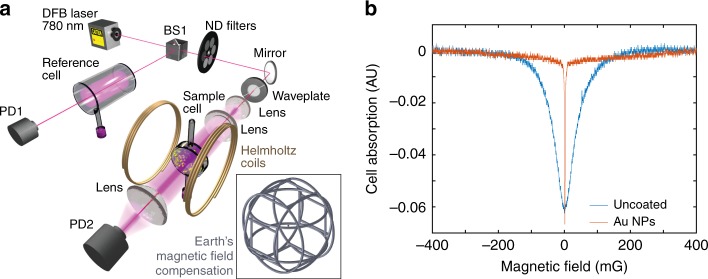
Comparison of electromagnetically induced transparency in the atomic cells. **a** Experimental setup for registration of EIT resonances. The reference cell in the first branch is used to fix the DFB laser frequency to the atomic absorption line maximum. The sample cell is studied in the second branch, situated in homogeneous magnetic field, linearly scanned around its zero value with a pair of Helmholtz coils. The sample cell and the entire setup is surrounded by three pairs of Helmholtz coils (~3 meters in diameter) compensating the Earth’s magnetic field, shown in the inset. **b** Electromagnetically induced transparency in Hanle-configuration, obtained in the ODA-Au-NPs coated cell (red line) compared to the one from the uncoated cell (blue line). The cell is illuminated by a 780 nm, circularly polarized laser beam with diameter completely covering the cell diameter

We tested our ODA-Au-NPs coating material by measuring the width of the magnetically sensitive Electromagnetically Induced Transparency (EIT) resonance in Hanle-type configuration^53,54^. Figure 6 shows the experimental setup used to investigate the anti-relaxation properties of the coating. The 780 nm distributed feedback (DFB) laser is tuned over the full frequency range of the Doppler-broadened D_2_ absorption line of Rb. The DFB laser beam is split, deflecting some of the light towards photodiode 1 through an off-the-shelf reference cell filled with Rb, in a natural mixture of isotopes. The rest of the beam is guided through the Au NP coated cell and the Rb absorption spectrum is monitored at photo diode 2. Next, the laser is frequency stabilized to the maximum absorption of the transition, starting from F_g_ = 2 hyperfine ground-state. The light polarization is set to circular and the beam diameter is expanded to 10 mm to cover the entire cell diameter. The cell is situated in the center of a pair of Helmholtz coils with diameter of d = 20 cm. In this way, homogeneous magnetic field **B** is applied orthogonally to the laser beam direction. The amplitude of **B** is linearly scanned in ranges up to ±1.7 G. The entire setup (and the sample cell) is situated in the center of three pairs of Helmholtz coils (~3 meters in diameter), compensating all 3 Cartesian components of the Earth’s magnetic field. This 3D Helmholtz setup produces highly homogeneous magnetic-field-compensation in the volume of our experimental setup. This volume is free of any ferromagnetic objects to prevent sources of stray magnetic field. The light transmitted through the Rb cell is then focused on PD2 and monitors the cell absorption.

The results show no sign of permanent magnetic moment on the Au NPs—the obtained resonance width is 33.5 times narrower compared to the EIT from uncoated cell. In fact, the resonance is of the same order of magnitude as one of the most established coatings—Octadecyltrichlorosilane (OTS)^55^. Moreover, our results are in agreement with several reports in the literature^56–58^ stating that the weak N-Au bond in amines-capped Au NPs does not lead to the creation of unpaired spins; the experiments therein detected no permanent magnetic moments.

The question of magnetism in Au NPs is the subject of intense debate. According to Nealon et al.^52^, “upon looking at all of the literature, one can only be surprised by the obvious lack of reproducibility”. Moreover, the same authors state that “It seems difficult to observe this magnetism with techniques other than SQUID magnetometry”. Within this context, our coatings combined with EIT measurements present a new opportunity to study the magnetism in plasmonic NPs. Whereas no net magnetic moment is observed in the case of ODA capping, other molecules, in particular thiol-based ones, could yield a different result.

In conclusion, we have presented a type of optical coating for alkali-metal vapor containers. Our coating allows fast and reproducible external control of the vapor density and related optical depth, crucial for quantum optics in these confined geometries. In this proof of principle, it was demonstrated that illuminating our coating significantly outperforms conventional LIAD and is compatible with standard polymer coatings used to preserve quantum states of single atoms and coherent ensembles. Further improvements of our coating are possible by tuning particle size, material composition and polymer environment. The coating can find applications in various containers, including optical cells, magneto-optical traps, micro cells, capillaries, and hollow-core optical fibers.

## Supplementary information

Supplementary Information

## Data Availability

The datasets generated and analyzed during the current study are available in the University of Bath repository, 10.15125/BATH-00529.
