# Supplementary material for: Atomic dispensers for thermoplasmonic control of alkali vapor pressure in quantum optical applications
Source: Nat Commun. 2019 May 24;10:2328. doi: 10.1038/s41467-019-10158-4 (PMC6534619; doi:10.1038/s41467-019-10158-4)
Supplement: Supplementary file 1 — Supplementary Information [file 41467_2019_10158_MOESM1_ESM.pdf]

Supplementary Information – Atomic dispensers for thermoplasmonic control of alkali vapor pressure in quantum optical applications

K. Rusimova et al.

Supplementary Note 1: Calculating quantum yields of desorption

We take the scanned Doppler profile (Figure 3(A)) and we measure the absorption amplitude δ. This value corresponds to the number of Rb atoms in thermal equilibrium. We differentiate numerically the desorption trace recorded in Fig. 3(C) to obtain the first derivative of the atomic density $dn(t)/dt$. The maximum desorption occurs at $t = 0$, as seen in Supplementary Figure 1. Thus, we obtain the initial desorbing rate $R = \frac{1}{\delta_0}\frac{dn(0)}{dt}$. Next, we perform a linear fit with zero intercept on the data in Fig. 4(A). By taking into account the geometry of the cell, we can calculate the number of desorbed atoms per incident energy as $\eta = aLn_0$, where a is the gradient of the fit and L = 0.125 cm is the characteristic length of the cell, and $n_0 = 5.6 \cdot 10^9$ atoms/cm^3 is the Rb vapor density in thermal equilibrium at 18 °C. Finally, we convert η to number of desorbed atoms per photon using $\gamma = \eta hf$, where h is Planck's constant and f is the frequency of desorbing light.

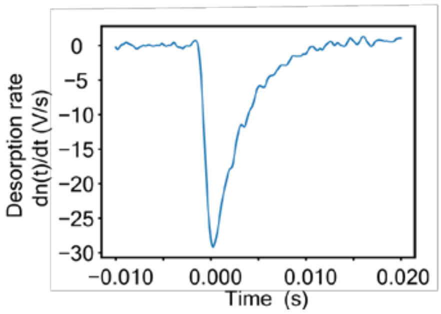

Supplementary Figure 1. Determining the maximum desorption. First derivative of the transmission signal plotted in Fig. 3(C). This derivative corresponds to the rate of desorption in units of V/s. To obtain the rate in units of Hz, we multiply by 1/δ_0, where δ_0 is the initial depth of the Doppler profile, measured in units of V, on the oscilloscope screen.

Supplementary Note 2: Vacuum cells

The vacuum cells were purchased from Precision Glassblowing Inc. and were fabricated of Pyrex. Their main body is cylindrical, with a 12.7 mm outer diameter and thickness of 1.6 mm. The cell windows are 5 mm apart. The two filling stems have an outer diameter of 3 mm and wall thickness of 0.6 mm. Rb filling was also performed by Precision Glassblowing Inc. The uncoated cell was baked at 425° C and 10-8 Torr for a few hours, whereas the coated cells were baked at the same pressure, but at 120° C prior to filling. After filling, the stems were sealed at 4 cm from the main bodies of the cells. All experiment were performed within the 6 months of the Rb filling.

Supplementary Note 3: Synthesis of Au NPs FPC448-FPC450

Octadecylamine-capped Au nanoparticles (4-7 nm in size) were synthesised at the Chemistry Department of the University of Bath following the procedure from reference.[1-4] A solution of $N(C_8H_{17})_4Br$ (365 mg, 0.65 mmol) in toluene (25 mL) was added dropwise to a vigorously stirred orange solution of $HAuCl_4 \cdot 3H_2O$ (112 mg, 0.28 mmol) in deionised water (25 mL) at room temperature. Both phases were strongly coloured (orange/red for the organic phase and orange for the aqueous phase) and when the aqueous phase became colourless, octadecylamine (842 mg, 3.12 mmol) was added as a solution in toluene (25 mL). The aqueous phase became milky and a solution of $NaBH_4$ (165 mg, 4.36 mmol) in deionised water (25 mL) was added to the stirred mixture. The organic phase became brown/black then

quickly turned deep purple as the aqueous layer turned colorless. The reaction was stirred for a minimum of 12h then the reaction layers were separated. The organic phase was concentrated under vacuum to a volume of 5 mL. The nano-particles were precipitated by adding absolute EtOH (350 mL) at room temperature. The mixture was then cooled at -60 °C and kept at -80 °C for 24h. When returned to room temperature, the supernatant EtOH was decanted and the dark purple precipitate was filtered on a 0.45 μm cellulose film, washed twice with EtOH and dried to afford 137 mg of dry product.

Supplementary Note 4: Cell coating procedure

All glass cells were first rinsed with DI water, blow-dried with dry N2 and baked at 80 °C in a furnace for 3 days. Two of the cells were functionalised with 1 M NaOH for 1 hour, followed by rinsing with DI water for another hour. The cells were then blow dried with N2. The main body of the cells and the first 4 cm of each stem were filled with a chloroform solution of the Au NPs (6 mg ODA-capped Au/ 6 ml chloroform) for up to 10 min. The cells were dried up again in a furnace at 80 °C for a day. The last two steps of the procedure were repeated.

Poly(dimethylsiloxane), bis(3-aminopropyl) terminated (PDMS) was purchased from Sigma Aldrich and was diluted into a 0.5 % diethyl ether solution by mixing for 1 hour in a magnetic stirrer. One of the Au NP coated cells and one empty cell were filled with the PDMS solution. They were then dried out by placing initially onto a hot plate at 80° C for half an hour and then into a furnace at the same temperature for a day.

Supplementary Note 5: UV-Vis spectroscopy

The UV-vis spectrum was recorded on a dilute solution of the nanoparticles in hexane at room temperature with a commercial Applied Photophysics Chirascan. All spectra were recorded over the range of 300 nm – 1100 nm with a resolution of 1 nm. Particle concentration was adjusted to give absorption of 1 near 300 nm for a 1 cm quartz cuvette. The spectrum was corrected for hexane absorption.

Supplementary Note 6: Transmission electron microscopy

Transmission electron microscopy (TEM) was performed at the Microscopy and Analysis Suite at the University of Bath on a Jeol 2100 Plus TEM. The samples were prepared by evaporating a drop of a dilute chloroform solution of the ODA-capped Au NPs onto a formvar coated Cu TEM grid. The TEM images were acquired at 200 keV accelerating voltage and × 200, 000 magnification.

Supplementary Note 7: Atomic force microscopy

A microscope slide was first finctionalised with 1 M NaOH solution for an hour, followed by rinsing with DI water for a further hour. The slide was then allowed to dry inside a fume cupboard for an hour, before being dipped into a dilute chloroform solution of the ODA-capped Au NP for 5 min. Finally, the slide was dried out in a furnace at 80 °C overnight. The atomic force microscopy (AFM) experiments were carried out using a Multimode Scanning Probe Microscope (Veeco, Plainview, NY) with a Nanoscope IIIA controller. Images were obtained in contact mode under ambient conditions.

Supplementary Note 8: Dark field optical microscopy

Microscopy images of the inner walls of the glass cell windows were obtained on a commercial Zeiss Axio Imager M2m wide-field microscope, with a halogen lamp for illumination. Images were taken in dark-field reflective mode, through a Zeiss Epiplan-NEOFLUAR 5x/0.15 HD objective, using an Axiocam 105 color camera.

Supplementary Note 9: Rubidium spectroscopy

A DFB laser from Toptica photonics, model DL-0780-0080-DFB-1, was used to scan the Rb absorption line. The photodiodes depicted schematically in Fig. 2 are an OPT101 Monolithic Photodiode and a PIN-photodiode. The reference cell used to check the stability of the laser has a length of 45 mm and a window diameter of 12 mm.

The source of 532 nm laser light was a Diode Pumped Solid-State Laser module based on a Nd-doped crystal. Although measures were taken for cleaning the output from the unwanted infrared (IR) radiation, we observe that a non-negligible portion of 808 nm, and 1064 nm still occurred mixed with the green light. We cleaned the unwanted residual IR radiation completely by passing the beam through a 60° dispersion prism made of heavy glass. Once clean, the green beam was aligned to the correct optical path with a pair of front-coated silver mirrors.

The desorbing laser beams were cut using a home-made optical shutter with a sharp metallic blade. The observed slope of blocking/releasing the laser beams were less than 1 ms and depended slightly on the different beam diameters. The shutter was controlled by a function generator (Zopan POF-10), producing individual pulses with controlled duration as well as periodically repeated pulses with controlled duty cycle.

The laser diodes were driven by SRS LDC 501 and Melles Griot Laser Diode Controllers, equipped with function generator SRS DS345 for sweeping the 780 nm-laser frequency around the Rb D_2 absorption line. The data acquisition was performed with GDS 1074B four channel oscilloscope with at least 100 kpts/screen.

Supplementary Note 10: Continuous illumination

Supplementary Figure 2 demonstrates the behaviour of the Rb vapour under continuous illumination, at 532 nm, over 5 s. It clearly illustrates that after the dramatic intial increase in the Rb vapour density, a steady state is reached after about 3 s illumination. This steady state is still much higher than the cold Rb vapour density. Moreover, the amplitude of the long-term steady state can be increased even further by optimising the cell geometry, e.g. by removing the filling stems. In addition, the equilibrium state can be maintained over longer periods of time by applying a stabilization process like the one in reference,[5] with the added benefit of a faster responce.

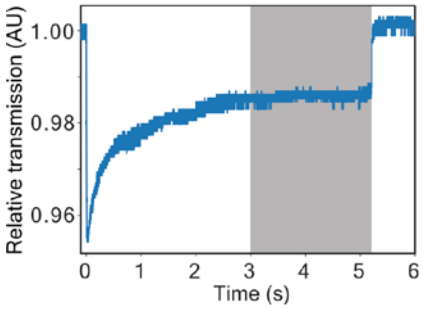

Supplementary Figure 2. Long term stability of Rubidium desorption. Rubidium desorption as indicated by the transmission (at 780 nm) through the Rb + Au NPs cell, under continuous illumination with 532 nm laser, for 5 s. The steady state of Rb vapor pressure is highlighted in grey.

Supplementary Note 11: Description of the EIT experiment

The method consists in orienting the Rb magnetic moment by optical pumping with circularly polarized laser light, while simultaneously applying homogeneous magnetic field orthogonally to the laser beam. The magnetic field value is scanned around zero and represented on the x-axis, while the laser beam absorption by the Rb atom is presented on the y-axis. The Earth's magnetic fields surrounding the cell was compensated by means of large 3D Helmholtz coils and no fero-magnetic objects in close proximity were allowed to avoid stray magnetic fields. Compared are the EIT resonances, obtained in the ODA-Au NPs coated cell (red line) and in the completely identical uncoated cell (blue line). At zero magnetic field the optical pumping is most efficient, bringing most efficiently the atoms to a magnetic sub-level not interacting with the laser light. At this point we observe the minimum of the absorption spectrum. In our case, the laser beam diameter covers the entire cell. The state of Rb magnetic moment orientation survives as long as the atom collides with the cell wall, where the interaction with the electromagnetic field of the bare glass surface destroys the light prepared orientation. A measure for the lifetime of the prepared magnetic moment orientation is the width of the EIT resonance. We clearly observe EIT narrowing of about 33 times in the coated cell as after the collision with the wall the atoms keep interacting with the laser being still prepared by the Zeeman optical pumping (as shown on Fig. 6). This is due to the elastic collision of the Rb atom with the C18-long hydrocarbon chain of the Au-capping agent (ODA). These molecular chains are approximately a few nanometers away from the gold nanoparticle itself[4,6,7] (~ 2 nm). Had there been a significantly permanent magnetic moment at the gold surface, it would have negated the preservation of the orientation on Rb atoms (i.e. scatter the electrons randomly across all Zeeman sublevels). Therefore, there is no evidence of magnetic field originating from our ODA-caped Au NPs.

Supplementary Note 12: Temperature equivalence

Supplementary Figure 3(a) illustrates the dynamics of the desorption process in the ODA-Au-NPs coated cells. As shown in Figure 1, the 1.5 mm in diameter beam from the DFB laser passes through the center of the investigated cell. The laser frequency is rapidly scanned across the ^{87}Rb (F_g=2) and ^{85}Rb (F_g=3) absorption lines for about 8 ms. PD2 is constantly monitoring the absorption signal. First, the amplitude (δ) of the ^{85}Rb (F_g=3) absorption is measured in the uncoated cell for different cell temperatures and represented with empty circles on the main plot. Next, the uncoated cell is replaced with the ODA-Au-NPs coated one and the absorption signal from PD2 is presented in the inset with blue line (light off), ^{85}Rb (F_g=3) maximum corresponding to the cell temperature (18.5 °C). Upon illumination with a 406 nm, 61 mW (46 mW/cm^2) light pulse, for 55 ms, the red line (light on) is recorded. The red line corresponds to the scan achieving maximal amplitude. This change happens in less than 20 ms after the abrupt application of desorbing light (slope < 1 ms). The red line allows us to estimate that the Rb vapor pressure achieved upon illumination corresponds to the vapor pressure achieved upon heating the cell to 45 °C (heating up the entire cell to that temperature). The measured equivalent temperature difference of ~27 °C is achieved in less than 20 ms. In addition, after the end of the desorption pulse, we observe that the vapor density drops back to the room-temperature value with almost the same time constant. It should be noted that the fast decrease of the Rb vapor density, is equally important, for instance in magneto-optical traps. Indeed, once such a trap is constructed, the remaining free atoms are disruptive and need to be quickly extracted from the vicinity of the trap. In our case, the rapid increase and decrease is observed at various desorbing laser wavelengths and is highly reproducible.

The data in Supplementary Figure 3(b) to Supplementary Figure 3(g) demonstrate that the Doppler width of the signal does not change significantly under 406 nm and under 532 nm illumination. Specifically, in Supplementary Figures 3(b) and (e), (i) represents the Rb

absorption spectrum line before the desorbing pulse, (ii) is the Rb absorption spectrum during the initial desorbing period, (iii) is the Rb absorption spectrum during the later desorbing period, as the Rb vapour pressure equilibrates. For clarity, these spectra are shown individually in Supplementary Figures 3(c) and 3(f). For comparison of their Doppler profiles they are shown normalized in Supplementary Figures 3(d) and 3(g). Clearly, there is no change of the Doppler width.

Also, please note that we performed the same verification for illuminations wavelengths 430 and 655 nm. The results are similar to those in Supplementary Figure 3 – no Doppler width change.

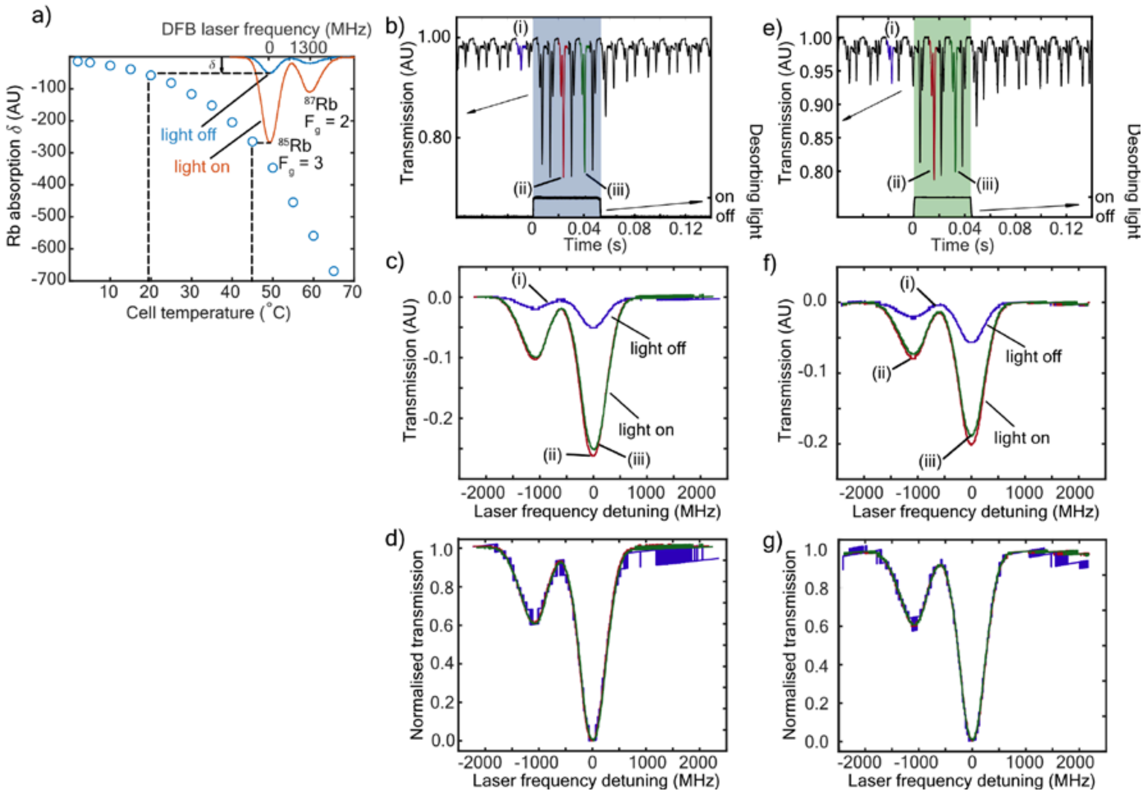

Supplementary Figure 3. Behavior of Doppler-broadened absorption profile. (a) Increase of the atomic absorption by a factor of ~5.4 in less than 20 ms. Main plot - amplitude (δ) of the Doppler-broadened Rb D_2 absorption spectrum, obtained from the uncoated cell when scanning the frequency of the DFB laser, at cell temperatures from 2 to 65 °C, is represented by blue empty circles. Inset - ODA-Au-NPs coated cell absorption with and without external illumination at 406 nm. (b) Rb absorption in the Au NP coated cell, with the laser frequency continuously scanned across the Rb D_2 absorption spectrum. The cell is illuminated with 406 nm laser light for 50 ms, as highlighted by the shaded region and the recorded shutter signal. (c) The Rb D_2 absorption spectrum centered at the F_g = 3 line for three selected regions from (b): (i) before the desorbing light pulse; (ii) at maximum Rb desorption during the light pulse; (iii) at lower Rb desorption during the light pulse, as the Rb vapour pressure equilibrates. (d) The same traces as in (c), but normalized. (e)-(g) Same as (b)-(d), but the Au NP coated cell is illuminated with 532 nm laser light.

Supplementary Note 13: Estimation of maximum vapour pressure achieved

In the monolayer coated cell the maximal absorption under light illumination is recorded and compared to the one obtained by simple heating of the uncoated cell (both cells having exactly the same geometry) (see Supplementary Figure 3). The light induced Doppler profile in the coated cell is obtained with the same maximum in an uncoated cell by heating it to about 45 °C.

For reference, we take the Rb vapor density from.[8] We also apply the vapor-pressure model given by.[9] We thus assess the light-induced Rb density in vapor pressure corresponding to 45 °C heated cell ($P_V^{illum} = 3.12 \cdot 10^{-6}$ Torr) and number density ($N^{illum} = 0.95 \cdot 10^{11}$ cm^{-3}) units. For comparison, these parameters in the coated cell, in darkness (experiment conducted at 18.5 °C) are $P_V = 0.19 \cdot 10^{-6}$ Torr and $N = 0.06 \cdot 10^{11}$ cm^{-3}. This corresponds to an increase of the vapor pressure of ~ 16 times in less than 20 ms. The slopes of both increase and decrease of the Rb vapor pressure are identical. Much higher magnitude of this factor is expected in cells without steams, in miniaturized cells and in hollow core optical fibers. Comparing the spectra of the Doppler profiles obtained in-dark-before- and under-illuminating the coating with desorption light we measure for ^{85}Rb F_g=3 the Doppler widths to be equal in dark and under illumination with the desorption pulse (about 560 MHz), for all desorption light wavelengths (406, 430, 532 and 655 nm).

Supplementary References:

1. Leff, D. V., Brandt, L. & Heath, J. R. Synthesis and Characterization of Hydrophobic, Organically-Soluble Gold Nanocrystals Functionalized with Primary Amines. *Langmuir* **12,** 4723–4730 (1996).
2. Shen, M., Du, Y., Hua, N. & Yang, P. Microwave irradiation synthesis and self-assembly of alkylamine-stabilized gold nanoparticles. *Powder Technol.* **162,** 64–72 (2006).
3. Yang, G.-J. *et al.* Preparation of glassy carbon electrode modified by hydrophobic gold nanoparticles and its application for the determination of ethamsylate in the presence of cetyltrimethylammonium bromide. *Sensors Actuators B Chem.* **128,** 258–265 (2007).
4. Qu, Q. *et al.* Open-tubular capillary electrochromatography using a capillary coated with octadecylamine-capped gold nanoparticles. *Electrophoresis* **29,** 901–909 (2008).
5. Bogi, a *et al.* Full control of sodium vapor density in siloxane-coated cells using blue LED light-induced atomic desorption. *Opt. Lett.* **34,** 2643 (2009).
6. de la Llave, E., Clarenc, R., Schiffrin, D. J. & Williams, F. J. Organization of Alkane Amines on a Gold Surface: Structure, Surface Dipole, and Electron Transfer. *J. Phys. Chem. C* **118,** 468–475 (2014).
7. Qu, Q. *et al.* Open-tubular gas chromatography using capillary coated with octadecylamine-capped gold nanoparticles. *Anal. Chim. Acta* **609,** 76–81 (2008).
8. Lide, D. R. *CRC Handbook of Chemistry and Physics*. *CRC Handbook of Chemistry and Physics* (CRC Press, 2001).
9. Steck, D. A. Rubidium 85 D Line Data. *Rubidium 85 D Line Data* (2013). Available at: http://steck.us/alkalidata . (Accessed: 20th September 2013)
